# The University and Hospital Bulletin

**Published:** 1904-10

**Authors:** 


### The University and Hospital Bulletin

DEVOTED TO THE INTERESTS OF THE UNIVERSITY OF BUFFALO
AND THE HOSPITALS.

EDITED BY
NELSON W. WILSON, M. D.

*Assistants:*
E. R. McGuire, M. D., Burton T. Simpson, M. D.

Under the auspices of the Faculty of the Medical Department of the University

M. D. Mann, A. M., M. D., Dean,
Charles G. Stockton, M. D.,
John Parmenter, M. D.,
Eli H. Long, M. D.,
Roswell Park, A. M., M. D., LL. D.,
Charles Cary, M. D.,
Herbert M. Hill, A. M., Ph. D.,
Herbert U. Williams, M. D.

AND

*Buffalo General Hospital* Henry Reed Hopkins, M. D.
*Hospital of the Sisters of Charity* Eugene A. Smith, M. D.
*German Hospital* Herman E. Hayd, M. D.

### Introductory.

IT SEEMS to me a happy decision on the part of the Buffalo Medical Journal that it has undertaken to publish with each issue, a Bulletin of the work done in the medical department of the University of Buffalo. It is an earnest of its own enterprise, as well as of interest in what is doing in Buffalo, and it should meet with a hearty response and with every encouragement from the alumni of the University.

The Medical Department has certainly been modest in placing before the profession of Western New York statements concerning the wide-spread importance of the work being done in the General Hospital, especially, as well as in the other hospitals of this city with which its teaching staff are connected, and it really is high time that more public notice were taken of the large amount of valuable material offered in the various institutions of this city.

Many men, in time past, have gone abroad to study medicine and found but little more over which to enthuse than can be found in the daily clinics in Buffalo. If the younger physicians of this city have not been as alert as they might be in taking advantage of that which is before them, as seems to be the case, they might well listen to some of the comments which are made by visitors from outside the city, or even from abroad, as to the amount of work done here and its relative value.

If now by a publication of this kind a better knowledge can be cultivated of what is going on, every reader of the Journal will be the gainer, and justice will at last be done to men who are spending their lives in the work. Every encouragement, then, should be given to this new enterprise.

ROSWELL PARK.

---

In Dr. Park's introductory to the first appearance of the University and Hospital Bulletin as a fixed department of the JOURNAL stress is laid on the clinical advantages of Buffalo. For several months past the JOURNAL has editorially called attention to the fact that the University of Buffalo, while not exactly lost sight of, was not thoroughly appreciated by the younger alumni. The underlying sentiment of these editorials was "Keep in Touch with Buffalo," and from them was developed the Bulletin as a department.

Primarily, the medical department of the University of Buffalo and the clinical work done by its faculty and teachers, will be kept well to the front with a view to establishing and maintaining rather more than a mere friendly interest in the institution. In connection with this work such news matters regarding the medical department as will be of general interest will be given a place; and, following the idea suggested by Dr. Park's remark concerning Buffalo's clinical advantages, narrative reports of work done at all hospitals in this city will be given a place in this department, preliminary to the reporting of cases in complete form by operators and clinicians.

---

### OPENING OF THE MEDICAL DEPARTMENT.

The medical department of the University of Buffalo will be opened Monday evening, September 26, with the usual formalities. The inaugural address will be delivered by Dr. Thomas H. McKee, of the University teaching staff, his subject being "Medical Education," which will be treated in a manner rather out of the ordinary. Dr. McKee is a forceful speaker, his ideas are well-balanced and the address will be unusually interesting to those who believe in the future of the university. Dr. McKee's paper will be published in the next issue.

---

### CHANGES IN THE PERMANENT FACULTY.

On September 12, at a meeting of the Council of the University, the first move toward the establishment of an arts department was made by a discussion concerning the establishment of University lectureships on literature, history, pedagogy and kindred subjects. The propositions made were tentative in character, and while nothing definite was established, the discussion has opened the way for more serious work in the near future along the lines of broadening influence and wider scope.

At the same meeting the resignation of Dr. John Parmenter as professor of anatomy was received and accepted, and Dr. Herbert U. Williams was elected a member of the permanent faculty to fill the vacancy. Dr. Williams was elevated to the professorship of pathology and bacteriology. Dr. Parmenter, while not now a member of the permanent faculty, remains on the university staff as professor of clinical surgery.

The work of the chair of anatomy will be carried on by Dr James A. Gibson, adjunct professor and demonstrator of anatomy. Dr. Gibson has been in charge of the anatomic room of the University for several years and there could be little improvement in his administration and methods of instruction.

Other matters of moment in connection with the extension of University influence are now under consideration of a committee from the medical faculty, consisting of Drs. Park, Stockton and Cary.

---

### THE DEGENERATION OF CUTANEOUS GROWTHS.

A case was referred to Dr. Park recently which indicates the danger of ignoring moles, warts and similar cutaneous excresences. Considerable has been written of late on the advisability of early removal of all such growths, even when they are apparently innocent in character, unimportant in size, and not at all annoying or inconvenient as regards location. Both Dr. Park and Dr. Keen have been preaching against the dangers of these long-considered benign growths which, while they may remain quiescent for years or even a life-time and never break down, too often do undergo transformation into malignancy.

The case referred to admirably illustrates such a condition and at the same time points to the wisdom of the attending physician who was sufficiently wideawake to recognise the first sign of danger and hurry his patient to operation, a procedure which in all probability saved her life. The patient is between 35 and 40, and had for years on the outer surface of the left arm a growth which had always been considered a plain mole. Of recent years it had shown signs of irritation. This became so marked a short time ago that her physician was consulted. He learned by careful questioning that the growth has changed in character as regards elevation, color and irritability and advised immediate operation for its removal. Dr. Park found the growth to be a well marked melanotic sarcoma with extensive axillary involvement. The tumor was removed by means of wide incision and the axilla cleaned out. This case is similar to several which Keen recently reported in which long neglect was followed by general infection and death.

---

A rather unusual and interesting case occurred on the service of Dr. Hayd at the German Hospital which demonstrates that the surgeon is often justified in operating in apparently hopeless cases even when the patient is apparently at the point of death.

A boy of 9 was received at the hospital with a history of stercoraceous vomiting for 48 hours. His condition was alarming—pulse 136, his skin cold and clammy and extremities blue. There was considerable abdominal distension but not much tenderness upon pressure. The stomach was washed out until the siphonage returned clear, when the patient was prepared and operated. A median incision was made and the intestines came into the wound greatly distended. They were followed until a band was found embracing one of the coils of the ileum, evidently an adhesion resulting from a previous appendicitis. The band was severed and the abdomen closed as rapidly as possible with through and through silkworm sutures. The following day the patient had two stools and went on to a perfect recovery, leaving the hospital on the seventeenth day.

This illustrates that even in the most desperate cases which are apparently without hope, when too much surgery is not necessary, recovery will often take place, and that operation for true obstruction of the bowels should be undertaken, even if the prognosis is most discouraging, because in many instances the cause is found to be merely a small band of adhesion.

---

Dr. Park's surgical clinics have begun at the General Hospital.

---

ARACHNO-ABDOMINAL DRAINAGE FOR HYDROCEPHALUS.

An operation was recently done by Dr. Marshall Clinton, instructor in surgery, for the relief of hydrocephalus, which a hasty review of the available literature places on record as the second of its kind.

The patient is a child and the operation was an arachno-abdominal drainage, briefly outlined as follows: A median abdominal incision was made, the child practically eviscerated and anastomosis made between the subarachnoid space and the abdominal cavity. During the operation the patient passed into a state of shock. Ten c.c. of normal salt solution and three drops of adrenalin were injected into the spinal cavity and immediate improvement followed. Drainage from the subarachnoid space to the abdominal cavity was established for forty-eight hours with a soft catheter. At the present time the child is improving and the hydrocephalic condition has been markedly benefited. Dr. Cushing, of Baltimore, has reported a similar operation. Dr. Clinton's report of the case will be published in the clinical department of the JOURNAL when the case is completed.

---

### TWO UNUSUAL GALLSTONE CASES.

Dr. Eugene A. Smith, adjunct professor of clinical surgery, will later report two rather unusual gallstone cases—unusual, because both were nursing mothers and both were received within a month. The first was a woman of 40, the mother of four children, the youngest between three and four weeks old when the mother entered the hospital in July. She gave a history of colicky attacks referable to the right side and covering a period of two years. Various diagnoses covering appendicitis, floating kidney and gallstone have been made. The first recent attack was a week after confinement, followed by four attacks within the succeeding three weeks. These attacks increased in severity and required from ¼ to ½ grain of morphine to ease the pain, which she described as "running through to the back." There were no chills, no jaundice. A diagnosis of biliary colic was made. The abdomen was opened and the gallbladder was found full of stones, two of which were impacted in the cystic duct. It was impossible to crush or work backward into the bladder these stones which seemed to be as large as hickory nuts, but which were, on subsequent removal, found to be somewhat smaller. Dr. Smith followed original lines in completing the operation, by splitting the gallbladder and duct down to the stones, ligating the duct in two sections, placing a rubber tube in the cystic duct for drainage of the duct, with a trough of underlying gauze acting as a floor for the tube to the surface, and an overlaying gauze drain, acting as a roof. The patient had a free flow of bile, but no complications; the gauze and tube were removed within two weeks and she went home on the twenty-eighth day, the wound not quite closed, but with no trace of bile. After the second week she took freely of nourishment and nursed the baby.

The second case came to operation within a month and the same general procedure was followed. This mother was at the time nursing a five months' baby. At the end of three weeks the child was brought to her for nursing twice a day. Other necessary feedings were by bottle.

